# Activin‐A Regulates Bone Morphogenetic Protein Signaling in Pulmonary Endothelial Cells Without Affecting Bone Morphogenetic Protein Type‐II Receptor Expression

**DOI:** 10.1002/pul2.70095

**Published:** 2025-05-05

**Authors:** Benjamin J. Dunmore, Nobuhiro Kikuchi, Wei Li, Paul D. Upton, Nicholas W. Morrell

**Affiliations:** ^1^ Victor Phillip Dahdaleh Heart and Lung Research Institute University of Cambridge Cambridge UK; ^2^ Department of Medicine, School of Clinical Medicine University of Cambridge Cambridge UK; ^3^ Department of Cardiovascular Medicine Tohoku University Graduate School of Medicine Sendai Japan

## Abstract

Activin‐A is elevated in pulmonary arterial hypertension (PAH) patients, and reportedly suppresses BMPR‐II. This suggests one mechanism of action for PAH drug, sotatercept, an activin‐ligand trap. However, we were unable to confirm that activin‐A reduces BMPR‐II in pulmonary endothelial cells. Thus, it seems unlikely that sotatercept influences BMPR‐II or PAH via this mechanism.

## 1 Introduction

Activins and bone morphogenetic proteins (BMPs) are ligands of the transforming growth factor‐β (TGF‐β) superfamily, which signal via heteromeric cell‐surface complexes of specific type‐I, activin receptor‐like kinase 1–7 (ALK1–7), and type‐II receptors [1]. Activins induce Smad2/3‐mediated signaling via the activin type‐II receptor (ACTR‐IIA) and ALK4/7, whereas in the endothelium, BMPs induce Smad1/5/9‐mediated signaling via the BMP type‐II receptor (BMPR‐II) and ALK1 [1].

Pulmonary arterial hypertension (PAH) is a devastating disease associated with progressive remodeling of vascular cells including the endothelium in the pulmonary circulation [1]. Without treatment, survival at 5 years after diagnosis is ~34% [1]. Genetic deficiency of BMP signaling is causative of disease, with diminished BMPR‐II observed in heritable and nonheritable cases and nongenetic animal models [1]. Conversely, increased circulating activin‐A levels are observed in PAH patients and lungs of mice with hypoxia‐induced PH [2]. Although vasodilator therapies have improved survival, these do not impact pathological pulmonary vascular remodeling [3]. Efforts have concentrated on developing novel therapeutics that rebalance the activin and BMP pathways. An activin‐A ligand trap, ACTR‐IIA‐Fc, demonstrated significant efficacy in experimental PAH animal models and a human ACTR‐IIA‐Fc therapy, sotatercept has successfully improved clinical endpoints and is approved for the treatment of PAH [4, 5, 6].

The precise mechanism of action of sotatercept in PAH remains uncertain. One proposed hypothesis is that activin‐A blockade restores BMPR‐II signaling. Of relevance, exogenous activin‐A treatment of pulmonary artery endothelial cells (PAECs) was reported to downregulate BMPR‐II protein expression, Smad1/5 phosphorylation and *ID1* gene expression after 6 h [7]. Although we previously saw no effect of activin‐A on Smad1/5 phosphorylation after a relatively short stimulation [8], we sought to clarify the effect of activin‐A treatment on BMPR‐II protein and downstream signaling in pulmonary endothelial cells.

## 2 Methods

### 2.1 Cell Culture

PAECs or pulmonary microvascular endothelial cells (PMECs; Promocell or Lonza) were maintained in Endothelial Cell Growth Medium (ECGM)‐2 plus 2% fetal bovine serum (FBS) or ECGM‐MV2 plus 5% FBS, respectively, including supplement mix and antibiotic‐antimycotic (penicillin, streptomycin, and amphotericin B; Invitrogen). For experimental studies, cells were either treated in low serum (0.1% FBS and A/A), or PAECs in supplemented ECGM‐2. Cells were treated with 20 ng/mL of activin‐A (R&D Systems) for 1, 6, and 24 h and recombinant human BMP9 (R&D Systems) at 0.3 ng/mL for 6 h, where indicated.

### 2.2 Immunoblotting

Cells were lysed in RIPA buffer (50 mM Tris‐HCl, pH 8, 150 mM NaCl, 1% Igepal, 0.5% sodium deoxycholate, 0.1% SDS, and 1x EDTA‐free protease inhibitor cocktail), and concentrations determined using the Bio‐Rad Lowry assay (Bio‐Rad Laboratories). Cell lysates (20–50 μg) were separated by SDS–PAGE and transferred to polyvinylidene fluoride membranes by semidry blotting (Cytiva). Membranes were probed with rabbit monoclonal antibodies phospho‐Smad1/5, phospho‐Smad2 (Cell Signaling), and phospho‐Smad1/3 (Abcam); rabbit polyclonal antibodies Smad1; Smad2 and Smad3 (Cell Signaling) or mouse monoclonal antibody against BMPR‐II (BD Transduction Laboratories). After washing, blots were incubated with secondary anti‐mouse/rabbit horseradish peroxidase antibody (Dako). Blots were reprobed with monoclonal antibodies for α‐tubulin or β‐actin (Sigma‐Aldrich). Densitometry performed using ImageJ software. Membranes were developed using enhanced chemiluminescence (Cytiva).

### 2.3 RNA Preparation and Quantitative RT‐PCR

Total RNA was extracted using the RNeasy Mini Kit with DNAse digestion (Qiagen). cDNA was prepared from ~1 μg of RNA using the High‐Capacity Reverse Transcriptase Kit (Applied Biosystems), according to the manufacturer's instructions. qPCR reactions were prepared in 384‐well plates using 50 ng/μL cDNA with PowerUp SYBR Green (Thermo Fisher Scientific) and primers at 200 nM. Primers for human: *BMPR2*, *ID1*, *ACTB*, *B2M*, *HPRT* were all designed using Primer3 (http://primer3.sourceforge.net/). Human *SMAD7* QuantiTect primers were purchased from Qiagen. Reactions were amplified on a QuantStudio 6Flex Real‐Time PCR system (Applied Biosystems). Target gene expression was normalized to the average of three housekeeping genes *ACTB*, *B2M* and *HPRT,* and the difference in the amount of product produced was expressed as a fold change.

### 2.4 Statistics

All data were analyzed using GraphPad Prism. Data are presented as mean ± SEM. Data were analyzed by Wilcoxon matched pairs *t*‐test, one‐way ANOVA with repeated measures or two‐way ANOVA, where indicated. *p* < 0.05 was considered significant.

## 3 Results

**Figure 1:**
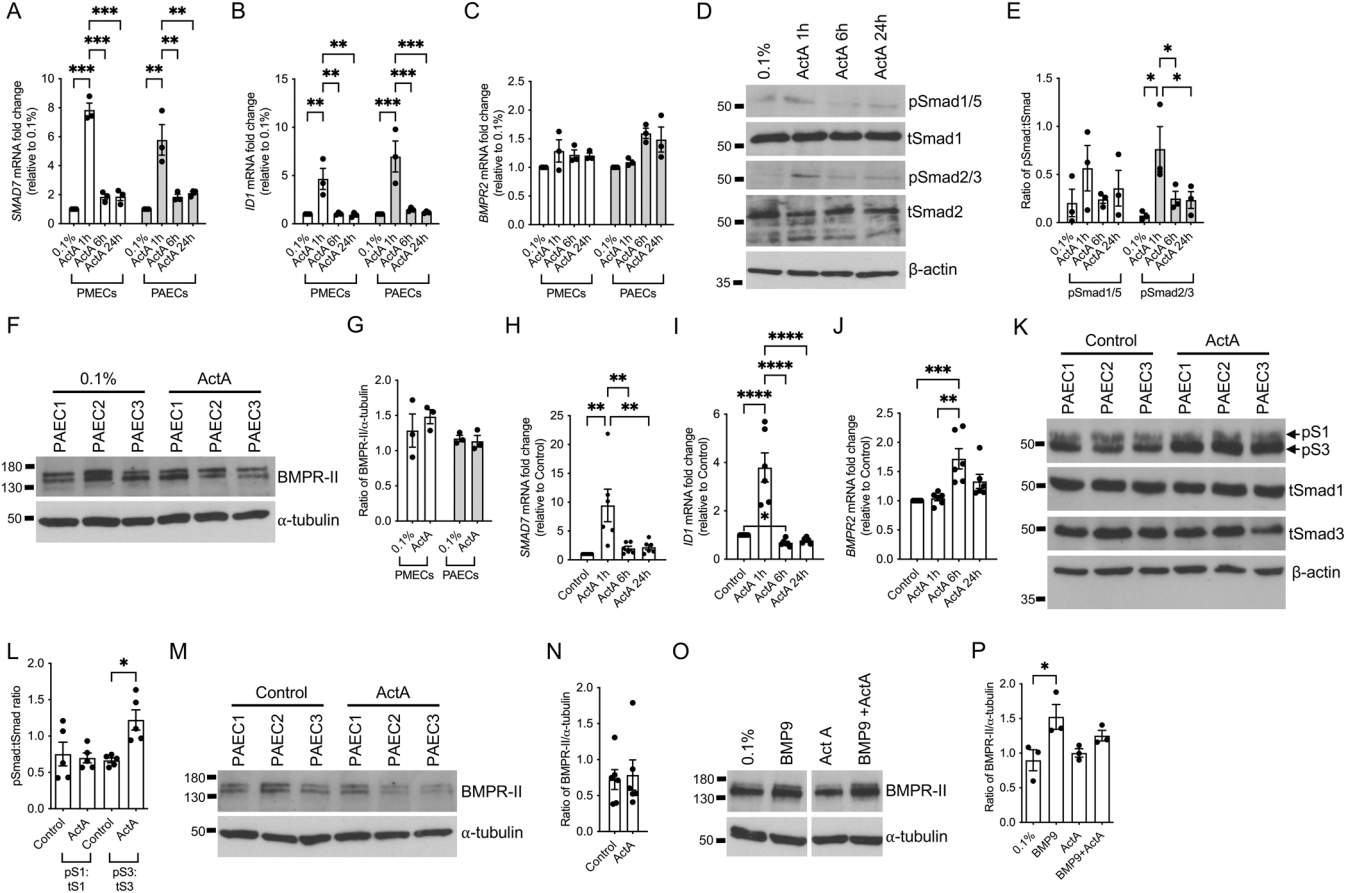
Effects of activin‐A treatment of BMPR‐II expression and BMP downstream targets in PMECs and PAECs in low serum and supplemented media conditions. (A–G) Human pulmonary microvascular endothelial cells (PMECs, *n* = 3) and human pulmonary artery endothelial cells (PAECs) were treated with activin‐A (20 ng/mL; ActA) for 1, 6, and 24 h in low serum (0.1% FBS) conditions, where indicated. RNA was isolated and *SMAD7* (A), *ID1* (B), and *BMPR2* (C) mRNA expression was assessed by normalizing to three housekeeping (HK) genes—*BACT*, *B2M*, and *HPRT*. (D) In PMECs, protein lysates were immunoblotted for phospho‐Smad1/5, phospho‐Smad2, total Smad1, total Smad2, and reprobed for β‐actin as a loading control. (E) Densitometry of the ratio between pSmad1/5 and total Smad1, and densitometry of the ratio between pSmad2/3 and total Smad2, both normalized to β‐actin. (F) In PMECs and PAECs, proteins were lysed after 6‐h ActA treatment and subsequently immunoblotted for BMPR‐II and reprobed for α‐tubulin as a loading control. (G) Densitometry of the ratio between BMPR‐II and α‐tubulin. (H–N) PAECs were treated with ActA (20 ng/mL) for either 1, 6, or 24 h in supplemented endothelial cell growth media, where indicated. RNA was isolated and *SMAD7* (H), *ID1* (I), and *BMPR2* (J) mRNA expression assessed by normalizing to three HK genes. (K) PAECs (*n* = 5) were treated with ActA for 6 h in supplemented media. Protein lysates were immunoblotted for phospho‐Smad3, using an antibody which cross‐reacts with phospho‐Smad1. Protein lysates were also immunoblotted for total Smad1 and total Smad3 and reprobed for β‐actin. (L) Densitometry of the ratio between pSmad1 and total Smad1, pSmad3 and total Smad3, normalized to β‐actin. (M) PAECs (*n* = 6) were treated with ActA for 6 h in supplemented media. Protein lysates were immunoblotted for BMPR‐II and reprobed for α‐tubulin. (N) Densitometry of the ratio between BMPR‐II and β‐actin. (O) PAECs (*n* = 3) were treated with or without Act‐A (20 ng/mL) and/or BMP9 (0.3 ng/mL) for 6 h in 0.1% FBS. Protein lysates were immunoblotted for BMPR‐II and reprobed for α‐tubulin. (P) Densitometry of the ratio between BMPR‐II and α‐tubulin. Two‐way ANOVA (A, B, and E). One‐way ANOVA (H, I, J, L, and P). Wilcoxon matched pairs test (I). *p ≤ 0.05, **p ≤ 0.01, ***p ≤ 0.001, ****p ≤ 0.0001. Error bars represent mean ± SEM.

As activin‐A was reported to negatively regulate the BMPR‐II pathway [7], we examined its effects on BMP signaling target genes (*SMAD7*, *ID1,* and *BMPR2*) and BMPR‐II protein expression, using PMECs and PAECs in low serum conditions [9]. Of note, activin‐A has previously been shown to increase *SMAD7* expression [10]. *SMAD7* and *ID1* were transiently elevated at the 1‐h timepoint (Figure 1A,B). *BMPR2* mRNA expression was not changed throughout the timecourse (Figure 1C). We observed no significant changes in Smad1/5 phosphorylation in PMECs, in contrast to the expected Smad2 phosphorylation by activin‐A (Figure 1D,E). Importantly, in three independent cell lines, no change in BMPR‐II protein levels was observed in either PMECs or PAECs treated with activin‐A for 6 h (Figure 1F,G).

We questioned whether our culture conditions contributed to these discrepancies. In the previously referenced study, treatments appeared to be conducted in the presence of serum and/or growth factors, as Smad1 was phosphorylated at baseline [7]. In media supplemented with 2% FBS and growth factors, PAECs treated with activin‐A transiently increased *ID1* and *SMAD7* expression at 1 h (Figure 1H,I). Intriguingly, *BMPR2* transcription was significantly increased after 6 h (Figure 1J). As 6‐h activin‐A treatment was reported to reduce Smad1 phosphorylation, we examined Smad1 and Smad3 phosphorylation in PAECs [7]. Baseline Smad1 phosphorylation was already evident in PAECs cultured in supplemented media (Figure 1K). However, activin‐A treatment did not affect Smad1 phosphorylation, but as expected Smad3 phosphorylation was increased in five biological replicates (Figure 1K,L). We determined BMPR‐II protein expression in PAECs following 6‐h activin‐A treatment and observed reduced BMPR‐II protein expression in some of the biological replicates (Figure 1M). However, the overall change in protein expression in six biological lines was not significant (Figure 1N).

We also evaluated the direct effects of activin‐A and BMP9 on receptor expression [7]. Since 2% serum contains active levels of BMP9, which might induce BMPR‐II expression in the endothelial cell, PAECs were treated with activin‐A in 0.1% FBS with BMP9 for 6 h. We observed no significant changes in BMPR‐II protein expression with activin‐A alone (Figure 1O,P). As expected, BMP9 treatment elevated BMPR‐II expression (Figure 1O,P). Interestingly, the increase in BMPR‐II by BMP9 in the presence of activin‐A treatment was nonsignificant (Figure 1O,P).

We conducted this study to provide clarification regarding the regulation of BMPR‐II and BMP signaling by activin‐A in vitro. Unlike a previous report, we did not observe reduced BMPR‐II protein expression. Our previous research showed no inhibitory effect by activin‐A on BMP9 signaling in PAECs [7, 8]. In fact, we observed a transient increase in *ID1* and *SMAD7* gene expression, which is not unexpected as activin‐A causes Smad1/5 phosphorylation in myeloma cell lines, which is enhanced by BMPR‐II loss [11].

In the report where activin‐A reduces BMPR‐II protein levels, treatment appears to have been conducted in the presence of FBS and/or growth factors [7]. Therefore, could there be a synergistic effect of activin‐A with serum and other factors on the downregulation of the BMPR‐II pathway? Indeed, activin‐A increases VEGF‐A secretion in human umbilical vein endothelial cells and an extravillous trophoblast cell line [12, 13]. In addition, activin‐A reportedly induces FGF expression and capillary formation [14]. Contrastingly, BMP9 inhibits VEGF‐induced angiogenesis and FGF‐induced proliferation [15, 16]. It is clearly apparent that BMPR‐II and activin‐A pathways have distinctly opposite effects, but how other angiogenic and vascular‐specific factors influence these remains to be fully elucidated. Another consideration is the activin‐A concentration (20 ng/mL) used in this study and the previously conducted research [7]. In IPAH patients, serum activin‐A was around 800 pg/mL compared to ~500 pg/mL in healthy controls [2]. In a more recent report, serum activin‐A levels in idiopathic, heritable, or anorexigen‐induced PAH patients were 583.7 ± 46.5 pg/mL compared to 328.7 ± 11.4 pg/mL in healthy subjects [17]. Additionally, in malignant pleural mesothelioma activin‐A plasma levels in controls were 361.3 pg/mL compared to 562.0 pg/mL in patients [18]. It is therefore possible that the concentrations used in this study are high in the context of lung disease.

There is a clear limitation to our study as characterization of the effects of activin‐A on the BMP pathway was exclusively conducted in cell culture conditions. In fact, transgenic overexpression of the precursor to activin‐A, inhibin‐βA (*INHBA*), reduces BMPR‐II and Smad1 phosphorylation [7]. However, our data do not fully support the reported findings that activin‐A downregulates BMPR‐II protein expression and BMP signaling in vitro. These results highlight the need to focus on the complex process of balancing TGF‐β and BMP pathways in treating PAH.

## Author Contributions

Benjamin J. Dunmore designed, performed, and analyzed the experiments and wrote the manuscript. Nobuhiro Kikuchi performed experiments. Wei Li contributed to writing the manuscript. Paul D. Upton conceived the study, designed experiments, and contributed to writing the manuscript. Nicholas W. Morrell conceived the study and contributed to writing the manuscript.

## Ethics Statement

The authors have nothing to report.

## Conflicts of Interest

P.D.U. is a scientific advisor to Interact Bio Ltd. N.W.M. is a founder and CEO of Interact Bio Ltd. All other authors declare no conflicts of interest.
